# Inhibition-Related Cortical Hypoconnectivity as a Candidate Vulnerability Marker for Obsessive-Compulsive Disorder

**DOI:** 10.1016/j.bpsc.2019.09.010

**Published:** 2020-02

**Authors:** Adam Hampshire, Ana Zadel, Stefano Sandrone, Eyal Soreq, Naomi Fineberg, Edward T. Bullmore, Trevor W. Robbins, Barbara J. Sahakian, Samuel R. Chamberlain

**Affiliations:** a Computational, Cognitive and Clinical Neuroimaging Laboratory, Division of Brain Sciences, Imperial College London, London, United Kingdom; b Department of Psychiatry, University of Cambridge, Addenbrooke’s Hospital, University of Cambridge, Cambridge, United Kingdom; c Department of Experimental Psychology, University of Cambridge, Cambridge, United Kingdom; d Behavioural and Clinical Neurosciences Institute, University of Cambridge, Cambridge, United Kingdom

**Keywords:** Compulsivity, Disinhibition, Inhibition, OCD, Phenotype, Phenotyping

## Abstract

**Background:**

Obsessive-compulsive disorder (OCD) is a prevalent neuropsychiatric condition, with biological models implicating disruption of cortically mediated inhibitory control pathways, ordinarily serving to regulate our environmental responses and habits. The aim of this study was to evaluate inhibition-related cortical dysconnectivity as a novel candidate vulnerability marker of OCD.

**Methods:**

In total, 20 patients with OCD, 18 clinically asymptomatic first-degree relatives of patients with OCD, and 20 control participants took part in a neuroimaging study comprising a functional magnetic resonance imaging stop signal task. Brain activations during the contrasts of interest were cluster thresholded, and a three-dimensional watershed algorithm was used to decompose activation maps into discrete clusters. Functional connections between these key neural nodes were examined using a generalized psychophysiological interaction model.

**Results:**

The three groups did not differ in terms of age, education level, gender, IQ, or behavioral task parameters. Patients with OCD exhibited hyperactivation of the bilateral occipital cortex during the task versus the other groups. Compared with control participants, patients with OCD and their relatives exhibited significantly reduced connectivity between neural nodes, including frontal cortical, middle occipital cortical, and cerebellar regions, during the stop signal task.

**Conclusions:**

These findings indicate that hypoconnectivity between anterior and posterior cortical regions during inhibitory control represents a candidate vulnerability marker for OCD. Such vulnerability markers, if found to generalize, may be valuable to shed light on etiological processes contributing not only to OCD but also obsessive-compulsive–related disorders more widely.

Obsessive-compulsive disorder (OCD) constitutes a global public health concern (1, 2, 3) and has been estimated to affect 2% to 3% of the population worldwide (4,5). Family and twin studies have provided strong evidence of a heritable contribution to the disorder (6), yet attempts to identify specific genetic loci have met with only partial success. For example, particular single nucleotide polymorphisms regulating cortical (especially serotonergic and dopaminergic) neurotransmission have been implicated in OCD, but inconsistently and typically with individually small effect sizes (7). It has been proposed that such limitations may be overcome in the future by using intermediate biomarkers such as those combining imaging and cognition (8, 9, 10, 11). Fundamentally, OCD can be considered as the mechanistic end point of underlying psychological and brain processes (12). Intermediate-level, biologically grounded vulnerability markers for OCD are lacking. By identifying latent vulnerability markers (phenotypes) linked with underpinning psychological processes contributing to a range of related mental disorders, new insights may be gleaned into underlying causal mechanisms, including genetic ones, cutting across conventionally discrete obsessive-compulsive and related disorders (13,14).

In prior work, it was suggested that objective measures of loss of inhibitory control constitute candidate latent phenotypes for OCD (15). Deficits on neuropsychological tasks of motor inhibition, including the stop signal task (SST) (16,17), have been observed in patients with OCD versus control participants, as now also shown by a meta-analysis (18). These deficits have also been found in clinically asymptomatic first-degree relatives of patients with OCD in several studies (16,19), highlighting their potential value as intermediate phenotypic markers of vulnerability. Cortico-subcortical circuits have been centrally implicated in OCD symptomatology (20). While initial OCD models focused on the prefrontal cortex, recent data implicate other cortical regions and the cerebellum in their pathophysiology (11,21, 22, 23, 24). In a recent meta-analysis of the available functional imaging literature, OCD was associated with hypoactivation during inhibitory control tasks in the anterior cingulate cortex, anterior insula/frontal operculum, supramarginal gyrus, orbitofrontal cortex, and thalamus/caudate (25). The SST is contingent on frontal lobe integrity and activates a distributed neural network, including frontal but also posterior brain regions (26, 27, 28). This task has been found to exhibit abnormal activation in patients with OCD and their clinically unaffected first-degree relatives (29) and so may be valuable for addressing connectivity vulnerability markers of the disorder.

While functional imaging has been widely used to explore case-control differences in brain activation in OCD (16,21,25,30,31), subsequent research has also elicited differences in the functional connectivity between different cortical regions. In a meta-analysis of seed-based resting-state functional imaging studies, OCD was associated with hypoconnectivity between frontoparietal (executive), salience, and default mode networks (22). Using the Multi-Source Interference task, which examines aspects of cognitive control, a prior study found altered regional connectivities in patients with OCD compared with control participants, including in paralimbic, sensorimotor, and default mode networks (32). In a functional imaging study using an SST, (33), abnormal negative coupling was found in patients with OCD versus control participants between the inferior frontal gyrus and amygdala. Similar results were evident, but to a lesser degree, in first-degree relatives of patients versus control participants (33).

The aim of this study therefore was to examine brain dysconnectivity during response inhibition as a candidate latent vulnerability marker for OCD. We hypothesized that patients with OCD and their clinically asymptomatic first-degree relatives would exhibit reduced connectivity between frontal and posterior brain regions within the inhibitory control network.

## Methods and Materials

### Participants

Patients with OCD were recruited from a National Health Service treatment center in the United Kingdom. Each patient entering into the study gave permission for the study team to contact a first-degree relative (by preference this was a same-gendered, similarly aged sibling when possible). Healthy control participants were recruited using media advertisements. Participants provided written informed consent after having the opportunity to read the information sheets and ask questions of the study team. The study was approved by the Cambridge Research Ethics Committee.

All study participants participated in an extended clinical interview supplemented by the Mini International Neuropsychiatric Interview (MINI; DSM-IV/ICD-10 version) (34), the Montgomery–Åsberg Depression Rating Scale (MADRS) (35), and the National Adult Reading Test (36). The MINI version used identifies the following mental disorders: major depressive disorder, dysthymia, suicidality, manic episodes, panic disorder, agoraphobia, social phobia, posttraumatic stress disorder, alcohol dependence/abuse, substance dependence/abuse, psychotic disorders, anorexia nervosa, bulimia nervosa, generalized anxiety disorder, and antisocial personality disorder. The MADRS rates depressive symptoms, and the National Adult Reading Test estimates IQ. For patients with OCD, symptom severity was assessed via interview using the Yale-Brown Obsessive-Compulsive Scale (37).

Inclusion criteria across all groups were being of adult age, being right-handed according to the Edinburgh Handedness Inventory (38), and being willing to provide written informed consent. Exclusion criteria across all groups were the inability to tolerate scanning procedures (e.g., owing to history of claustrophobia), contraindication to scanning (e.g., metallic implant, pregnancy), current depression (defined as those individuals meeting DSM criteria on the MINI and/or those with an MADRS score >15), current mental health disorder on the MINI (except OCD in the OCD group), history of neurologic disorders (e.g., Tourette’s syndrome, tics, major head trauma), history of psychosis, and history of bipolar disorder. In the OCD group, participants were required to meet DSM criteria for the disorder based on clinical interview and the MINI, to have primarily washing/checking symptoms, and to have a Yale-Brown Obsessive-Compulsive Scale total score >16. Our rationale for including patients with mainly washing/checking symptoms was that washing symptoms in particular are extremely common in OCD (5), and we wished to include the same symptom-related criteria as in our previous case-relative-control behavioral study (19). Patients with OCD with clinically significant hoarding were excluded because hoarding differs from mainstream OCD and is now listed separately from OCD in diagnostic nosological systems (39). In the OCD relatives group and control group, participants were required to be free from history of OCD (including no clinically significant symptoms based on extended clinical assessment such as the MINI), to be free from other mainstream mental disorders (e.g., mood disorder, anxiety disorder), and to not be receiving psychotropic medication(s).

### Stop Signal Task

Participants completed pretraining on the SST (40) prior to functional magnetic resonance imaging (fMRI), with a view to minimizing between-group differences in behavioral measures that can confound interpretation of imaging connectivity data. Participants then completed the task during fMRI. We used a version of the task optimized for fMRI as described elsewhere (41). In brief, individuals viewed a series of left- and right-pointing arrows (the go signals) and were instructed to respond as quickly as possible by clicking the button with their right hand, depending on which direction the arrow was pointing. Intermittently, a down-pointing arrow (the stop signal) would appear on the screen for a variable time interval (initially 200 ms) after a go signal, and participants were instructed to stop their initiated response when it appeared. By modulating the go–stop gap as previously described, the task was designed for a 50% successful inhibition outcome and was performed by each participant for approximately 8 minutes. The stop signal reaction time was calculated using the simple/standard way for such designs, that is, by subtracting the mean go–stop interval from the mean reaction time. Scanner behavioral data recorded for each participant are presented in the Supplement, with analyses indicating that the task design functioned correctly [no behavioral differences between groups and *p*(inhibit) close to 50% in each group as expected].

### Functional Imaging Acquisition

Imaging data were acquired at the Wolfson Brain Imaging Centre at the University of Cambridge. Participants were scanned with a 3T Siemens TIM Trio scanner (Siemens Corp., Erlangen, Germany). While the participants were undertaking the SST, blood oxygen level–dependent sensitive three-dimensional volume images were acquired every 2 seconds. The first 10 images were discarded to account for equilibrium effects of T1. Each image volume consisted of 32 slices of 4 mm thickness, with in-plane resolution of 3 × 3 mm and orientated parallel with the anterior commissure–posterior commissure line. A standard echo-planar imaging sequence was used with 78° flip angle, 30 ms echo time, and temporal resolution of 1.1 seconds in a continuous descending sequence. The field of view of images was 192 × 192 mm, a 64 × 64 matrix, 0.51 ms echo spacing, and 2232 Hz/pixel bandwidth. In addition, a 1-mm resolution magnetization prepared rapid acquisition gradient-echo structural scan was collected for each individual with a 256 × 240 × 192 matrix, 900 ms inversion time, 2.99 ms echo time, and 9° flip angle. Scan preprocessing was conducted using the standard procedure in SPM12. Data for each participant were motion corrected, registered to the structural magnetization prepared rapid acquisition gradient-echo, spatially warped onto the standard Montreal Neurological Institute template using DARTEL toolbox, upsampled to 2-mm cubed voxels, and spatially smoothed using a Gaussian filter (8 mm full width at half maximum Gaussian kernel).

### General Linear Modeling Analysis

fMRI data were analyzed to determine blood oxygen level–dependent signal changes in response to participants performing the SST. General linear model analysis was applied at the individual participant level in SPM12. The data were high-pass filtered (cutoff period = 180 seconds) to remove low-frequency drifts in the MRI signal. Regressor functions for each condition were created by convolving timing functions indicating the onset of each of six event types, with a basis function representing the canonical hemodynamic response. The event types were successfully versus unsuccessfully inhibited left or right responses and the left or right responses in go trials. Six regressors were included representing rotations and translations for the x-, y-, and z-axes.

### Group-Level Analysis

Whole-brain maps depicting beta weights for the experimental predictor functions from the first-level models were collated for group-level analyses using a full-factorial 2 × 2 × 3 design, where outcome of the stop trials (successful inhibition or unsuccessful inhibition) and the direction with which the response was made were the within-subject factors and group (OCD, relative, or control) was the between-subject factor. The following four a priori voxelwise contrasts were estimated: 1) the positive effect of condition (*t* contrast of the mean of all stop trials vs. 0), which captures regions of the brain that are significantly active during stop trials; 2) successful minus failed stop trials; 3) the main effect of group; and 4) the group × condition interaction. To correct for multiple comparisons across the whole-brain mass, contrast images were thresholded at *p* < .05 voxelwise, and false discovery rate cluster correction was then applied at *p* < .05. Significant effects of group were further interpreted by fitting 5-mm-radius spheres at the peak coordinates of a given significant *F* test map and conducting post hoc permutation tests for each groupwise comparison (10,000 permutations per test).

### Regions of Interest

Regions of interest (ROIs) were generated by our in-house three-dimensional watershed transform algorithm (42,43). The method was used because it can accurately and efficiently decompose thresholded statistical activation maps into discrete clusters even when the clusters are contiguous. It was conducted at the group level based on the thresholded statistical maps to enable connectivity across the activated network to be examined. When generating the ROIs, the within-subject contrasts (1 and 2) were also thresholded voxelwise at *p* < .01 to focus on the most active brain regions. The ROIs formed the basis of the connectivity analyses.

### Connectivity Analysis

Measures of task-evoked network connectivity were estimated using psychophysiological interaction (PPI) models, which quantify how the correlation in activity between pairs of brain regions differs across task conditions. Notably, the classic PPI method focuses on a single task contrast across task conditions. More recently, a generalized form of PPI (gPPI) was developed that simultaneously assesses the impact on connectivity of multiple task conditions. We used a custom MATLAB (The MathWorks, Inc., Natick, MA) implementation of the following gPPI model:

$$Y^{T}=\;\beta_{0}\;+\;\left[Y^{S},H\left(X\right),E\right]\beta_{G}\;+\;\left[Y^{S}*H\left(X\right)\right]\beta_{j}\;+\;e,$$

where X was the matrix containing psychological time courses (i.e., time courses for encode, maintain, and probe events) and H(X) was the hemodynamic response function convolution of that matrix; $Y^{T}$ was the target time series and $Y^{S}$ was the source time series; E was the nuisance regressor matrix defined previously in the preprocessing stage; $\beta_{G}$ included weights of no interest and $\beta_{j}$ included the weights for the PPI predictors, which were the target of further analysis; $\beta_{0}$ was the intercept and ewas the residual error. This model was estimated for all pairs of connections to form a connectivity matrix, and upper and lower triangles were averaged to form an undirected weighted connectivity matrix for each condition in the design matrix. gPPI models included successful inhibition, failed inhibition, and go trials for each participant group. Two contrasts were generated: all stop signals minus all go trials and successful minus failed stop signal trials. Mixed analyses of variance were applied to test for significant differences among the three groups in the pattern of PPI estimates across ROIs. Pairwise *t* tests were then applied at an uncorrected *p* < .01 threshold to characterize the basis of any significant interactions.

## Results

In total, 20 patients with OCD, 18 of their nonsymptomatic first-degree relatives, and 20 control participants completed the study. The demographic and clinical features of the sample are presented in Table 1, where it can be seen that the groups were well matched on age, gender, and IQ. As expected, patients with OCD scored significantly higher on MADRS total scores than the other groups, but mean scores were well below the threshold for clinically significant depression, in keeping with the exclusion criteria used. Task-related behavioral measures did not differ significantly among the groups (see Supplement). The following numbers of patients were taking psychotropic medication: eight selective serotonin reuptake inhibition monotherapy and two selective serotonin reuptake inhibitor plus low-dose antipsychotic medication. One patient was also taking occasional lorazepam but had not taken this within 48 hours of study participation.

**Table 1:** Demographic and Clinical Characteristics of Patients With Obsessive-Compulsive Disorder, Their Unaffected First-Degree Relatives, and Healthy Control Participants

	Patients (*n* = 20)	Relatives (*n* = 18)	Control Participants (*n* = 20)	Statistic	*p*
Demographic Measures					
Age, years	37.6 ± 14.6	40.7 ± 10.8	36.3 ± 8.3	*F*_2_ = 0.7115	.4954
Gender, *n* (male:female)	20 (17:3)	18 (13:5)	20 (15:5)	χ^2^_2_ = 1.007	.6044
NART IQ	115.4 ± 5.2	114.6 ± 7.2	115.9 ± 6.2	Kruskal–Wallis statistic = 0.23	.8914
Clinical Measures					
MADRS	7.5 ± 7.5	2.33 ± 3.3	1.30 ± 3.4	Kruskal–Wallis statistic = 16.05	.0003
Y-BOCS obsessions	11.15 ± 2.58	–	–	–	–
Y-BOCS compulsions	11.85 ± 2.62	–	–	–	–
Y-BOCS total	22.50 ± 5.30	–	–	–	–

Values are presented as mean ± standard deviation unless otherwise stated. One-way analysis of variance or Kruskal–Wallis statistical tests were used depending on the normality of data.

MADRS, Montgomery–Åsberg Depression Rating Scale; NART, National Adult Reading Test; Y-BOCS, Yale-Brown Obsessive Compulsive Scale.

### Activation Results for the SST

**Figure 1:**
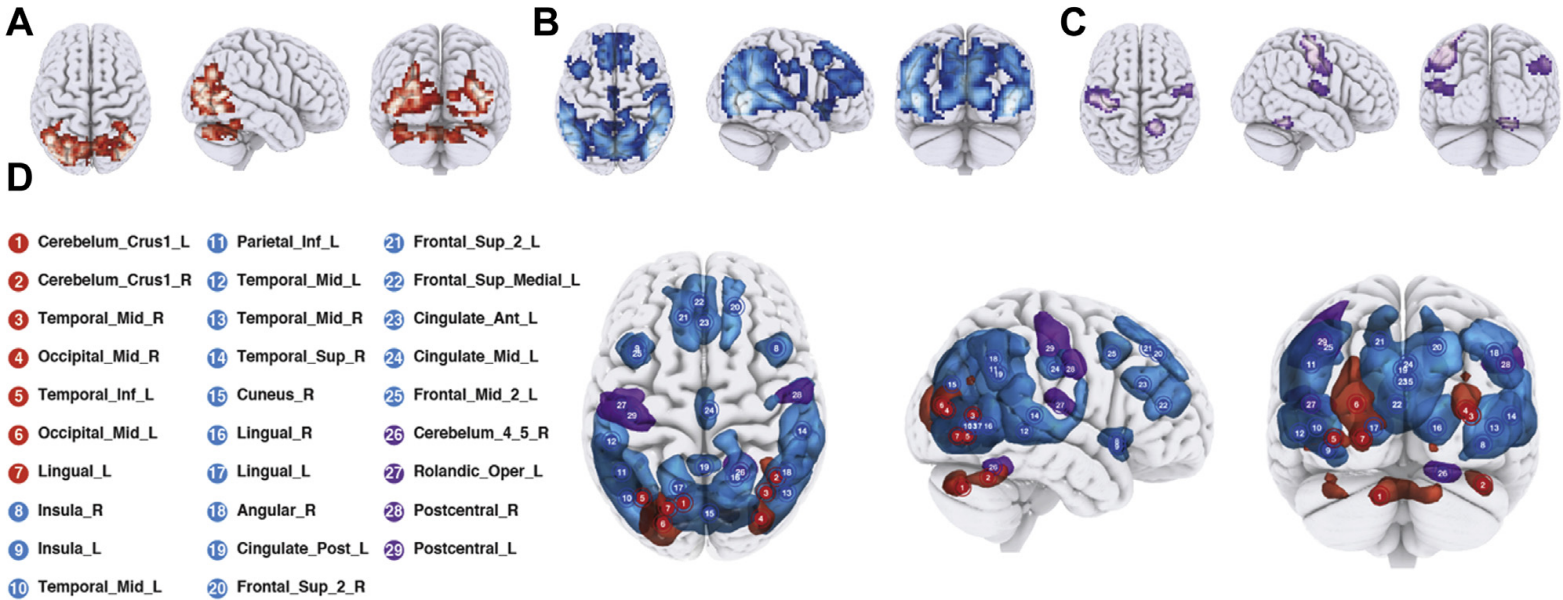
Significant activation maps for the contrasts of interest during the stop signal task. **(A)** Brain regions showing a main effect of group (false discovery rate *p* < .05). **(B)** Brain regions activated during stop signal trials (false discovery rate *p* < .05). **(C)** Brain regions underactivated for successful minus failed stops (false discovery rate *p* < .05). **(D)** Regions of interest for subsequent connectivity analyses on a brain map and also labeled, generated from the above contrasts and color coded in keeping with **(A)** to **(C)**. Ant, anterior; Inf, inferior; L, left; Mid, middle; Oper, operculum; Post, posterior; R, right; Sup, superior.

Activation differences for the SST contrasts of interest, along with the extracted ROIs, are summarized in Figure 1 (all false discovery rate *p*s < .05). There was a main effect of group (Figure 1A), yielding group differences mainly in the occipital lobes, specifically in the left and right occipital cortex (Brodmann areas 18 and 19), the temporal occipital fusiform cortex (Brodmann area 37), and the cerebellum. Post hoc permutation tests indicated that the group effect was due to hyperactivation in patients with OCD versus both other groups maximal in the bilateral lateral occipital complex (both *p*s < .001) (Supplemental Figure S1). Brain regions significantly activated during stop signal trials, across all participants, are shown in Figure 1B. It can be seen that the SST activated the distributed inhibitory control network, including the bilateral inferior frontal gyrus, insula, and anterior cingulate cortex. For the contrast of successful minus failed stops across all participants, relative hypoactivation was observed (Figure 1C) in regions associated with motor responses (including Brodmann areas 4 and 6). This is consistent with failed stops activating relevant motor areas owing to action as compared with there being no motor response for successful stops. The interaction of group × successful minus failed inhibition did not yield significant regions.

### Group Differences in Connectivity for the SST

**Figure 2:**
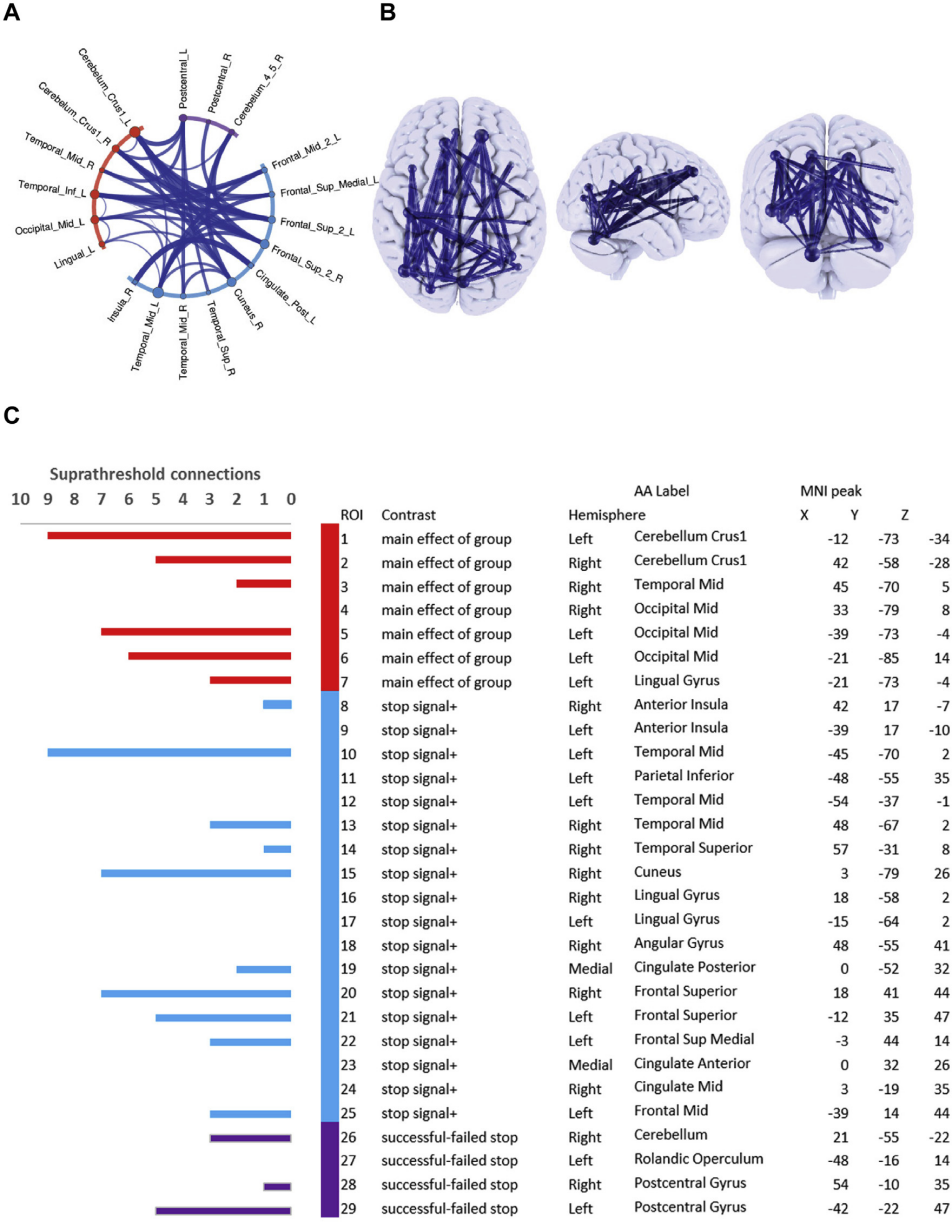
Results from connectivity analyses. **(A)** Schemaball showing abnormally hypoconnected regions in patients with obsessive-compulsive disorder and relatives versus control participants. Each region of interest (ROI) is indicated by a peripheral label. Curved lines within the circle indicate ROI–ROI connections that were significantly hypoconnected in patients and relatives versus control participants. Thicker curved lines indicate greater abnormality (mean psychophysiological interaction coefficient). The outer circumference of the circle is color coded to indicate the contrast of interest as per Figure 1, and the size of nodes on the peripheral circle represents the total number of suprathreshold abnormal connections (i.e., nodal degree). **(B)** Glass brain representation of abnormal connections from **(A)** to show anatomical extents. **(C)** List of all ROIs and the number of suprathreshold connections with other regions for each ROI. Color codings refer to the task contrasts of interest. AA, Automated Anatomical Labeling; Inf, inferior; L, left; Mid, middle; MNI, Montreal Neurological Institute; Post, posterior; R, right; Sup, superior.

The 29 functional ROIs from the above activation maps (Figure 1D) were used for the subsequent connectivity analysis. For the SST contrast (stop signal minus go trials), there was no significant main effect of group on gPPI connectivity (*F* = 0.69, *p* = .50), there was a significant effect of connection (*F* = 1.78, *p* = .011), and there was no significant interaction (*F* = 1.17, *p* = .19) (all Greenhouse–Geisser corrected). When applied to the success minus fail contrast, there was a significant main effect of group (*F* = 3.67, *p* = .032) and connection (*F* = 1.71, *p* = .016) and a significant interaction (*F* = 1.38, *p* = .041) (all Greenhouse–Geisser corrected). These results indicated that the task conditions affected network connectivity in different ways across the three groups. To characterize the basis of the effects at the node level, the coefficients were contrasted pairwise for patients and their relatives versus control participants, thresholded at *p* < .01 two tailed (Figure 2). A widespread pattern of reduced connectivity was evident in patients with OCD and their relatives. Summing the number of supra-threshold connections for each node highlighted a high degree of abnormality affecting cerebellum area crus 1 connectivity bilaterally, middle occipital gyrus bilaterally, superior frontal gyrus and superior medial frontal cortex, left middle temporal, and left postcentral gyri.

## Discussion

This study evaluated functional brain dysconnectivity during response inhibition as a candidate vulnerability marker for OCD. Consistent with our hypothesis, the key finding was that patients with OCD and their first-degree relatives had abnormally reduced functional connectivity during the SST between frontal and posterior brain regions, including the frontal cortex, occipital cortex, and cerebellum. These novel findings accord well with the notion that functional connectomics constitutes a candidate vulnerability marker for OCD, supporting neurobiological models of the disorder implicating loss of cortically mediated inhibitory control, not only constrained to the frontal lobes but also involving distant posterior brain regions (15,44).

Conventional analysis confirmed that the fMRI SST activated neural circuitry, including the bilateral inferior frontal cortex and anterior cingulate cortex as well as more posterior parts of the brain playing a role in visual attention streams (Figure 1). This is in keeping with prior lines of research implicating such regions in cortically mediated motor inhibition processes (26,45, 46, 47). We generated a set of ROIs using an innovative watershed algorithm to examine connectivity differences between groups using a gPPI model. This identified widespread patterns of hypoconnectivity, common to patients with OCD and their relatives, versus control participants in frontal and posterior brain regions (Figure 2). Overall group differences in connectivity during the SST were specifically detected during the success–fail contrast, with connectivity being lower in patients and relatives versus control participants. In the absence of significant overall stop–go differences in connectivity among the groups, this suggests that patients with OCD and their relatives had higher connectivity for failed stops and/or lower connectivity for successful stops compared with control participants. Ultimately, determining what this means on a process level requires further investigation examining causal dynamics. However, the implicated neural regions are likely to operate via mutual bidirectional connections to facilitate response inhibition (48). It is interesting to note that certain frontal brain regions found to be abnormally connected here during response inhibition (i.e., inferior frontal cortex/insula) were previously found to exhibit reduced striatal-related connectivity in OCD in association with cognitive rigidity (49).

The most commonly dysconnected nodes common to patients and their asymptomatic first-degree relatives included frontal cortical, occipital, and cerebellar regions (Figure 2). Conventional neurobiological models of OCD have focused on the frontal lobes, whereas the current findings implicate abnormal connections involving not only frontal brain regions but also these other brain regions. This is in keeping with several tiers of OCD research more broadly (21,24,50), including connectivity studies. For example, resting-state connectivity changes in OCD were maximal in the cerebellar crus 1 region (51), and machine learning algorithms designed to discriminate patients with OCD from control participants based on resting-state connectivity indicated important contributions from not only frontal regions but also occipital and cerebellar regions (52). To our knowledge, only one previous study has examined task-related functional dysconnectivity as a candidate vulnerability marker for OCD (53). This study found reduced functional connectivity between the right dorsolateral prefrontal cortex and the basal ganglia (putamen) during executive planning (53). Resting-state connectivity changes have also been described in the literature, in patients with OCD and their relatives, involving distributed brain regions (54,55). Collectively, the emerging evidence thus suggests important dysconnectivity not only between cortical and subcortical regions but also between anatomically distant cortical regions in OCD, findings that are likely to be contingent on the nature of the cognitive probe used to explore such neural circuitry.

In terms of group differences in SST-related brain activation (as opposed to functional connectivity), we found differences in posterior brain regions, maximal in the bilateral lateral occipital complex. This result was attributable to hyperactivation in patients versus both other groups, whereas activation in relatives did not differ from control participants in this region. There was no group × successful minus failed inhibition interaction, indicating that this abnormality was common to inhibition trials on the task whether or not inhibition was successful. The lateral occipital complex plays an important role in visual attentional processing, including representation and perception of objects (56) and faces (57). One interpretation of the current finding is that hyperactivation of this visual processing region may be related to hypervigilance in OCD or an expectation of an environmental threat. Owing to the unpredicted nature of this result, replication is required before firm conclusions can be made. Nonetheless, this result suggests that tasks designed to probe visual attentional streams may be valuable in OCD research.

Although this is the first study to address inhibitory control–related functional connectivity as a candidate vulnerability marker for OCD, several limitations should be considered. We recruited patients with primarily washing/checking OCD symptoms who did not have comorbidities. As such, it remains to be demonstrated whether the findings generalize to patients with other primary symptoms or to those who have comorbidities. Owing to the sample size, power may be limited. Our approach could be viewed as conservative because nodes of interest were generated using false discovery rate *p* < .05; hence, and in view of the sample size, some neural nodes implicated in OCD, but with a smaller effect size, may have been overlooked. Presupplementary motor activation abnormalities were previously found in patients with OCD and their relatives (29), but we could not replicate this finding in the relevant ROIs (see Supplemental Table S2). Likely because participants were pretrained, they did not differ on stop signal behavioral measures; this is an advantage because it simplifies imaging interpretation, but the corollary is that our study did not measure neural changes related to impaired inhibition but rather measured neural changes related to inhibition per se. Owing to the nature of the gPPI analysis, it could not be established whether there was heightened connectivity during go trials or decreased connectivity during stop trials in the patients and relatives. Our connectivity difference was in the contrast of successful–failed stop trials. Control participants showed heightened connectivity when stopping was successful relative to unsuccessful. In OCD, this effect was reduced. This is an interesting pattern of connectivity difference. Patients with OCD may be engaging the network more during unsuccessful stop trials, in line with abnormal post-error processing. Or, it may be that they engage the network less during the successful stop trials. The fact that we see this difference but no cross- group difference for stop–go suggests that it is both. This aspect could be assessed in future studies by including rest blocks, allowing activity and connectivity during routine responding to be estimated separate from the resting baseline. While some patients with OCD were receiving psychotropic medications, functional dysconnectivity was also found in these patients’ relatives who were not receiving any psychotropic medications. Hence, while we cannot address effects of such pharmacotherapies on connectivity owing to the sample size, our key findings were not due to such effects. Prior work found treatment-related changes in activation during a Stroop task, which examines attentional inhibition processes, in patients with OCD (58). Future work should examine effects of treatment on functional connectivity during inhibition tasks in OCD. We did not observe robust differences between the OCD and first-degree relative groups in functional connectivity. Identification of differences between these two types of group using larger samples in future work may be valuable to identify mechanisms associated with chronicity/instantiation of OCD as opposed to vulnerability toward OCD. Lastly, the current study focused on cortical functional connectivity; however, given the prominent role of the basal ganglia in OCD models, future work should also look at cortico-subcortical connectivity on the SST, with there already being evidence of abnormalities in OCD using an executive planning task (53).

In conclusion, we found that hypoconnectivity during response inhibition, involving frontal and posterior brain regions, may constitute a candidate vulnerability marker for OCD. Future studies could use such cognitive probe connectivity approaches to help delineate etiological factors involved in OCD and extend research into other obsessive-compulsive–related disorders (59).
