# Supplementary material for: Inhibition-Related Cortical Hypoconnectivity as a Candidate Vulnerability Marker for Obsessive-Compulsive Disorder
Source: Biol Psychiatry Cogn Neurosci Neuroimaging. 2020 Feb;5(2):222–30. doi: 10.1016/j.bpsc.2019.09.010 (PMC7003031; doi:10.1016/j.bpsc.2019.09.010)
Supplement: Supplemental Material [file mmc1.pdf]

Inhibition-Related Cortical Hypoconnectivity as a Candidate Vulnerability Marker for Obsessive-Compulsive Disorder

Supplemental Information

Stop-Signal Task Connectivity in OCD

Behavioural Measures

The behavioural data were statistically analysed in GraphPad Prism 7 (GraphPad Software, La Jolla California USA, www.graphpad.com). Behavioural results were tested for normality with the Shapiro-Wilk normality test. One-way ANOVA and Kruskal-Wallis tests were applied subsequently to test for significant differences (threshold $p<0.05$). SST task response times (RT) and stop-signal reaction times (SSRT; Table S1) were analysed across groups. Participants responded ~180 times during the task. Each participant was presented with 59 stop-signal cues, and all participants showed a high failure rate (controls (C) = 49%, relatives (R) = 53%, patients (P) = 53%). This is expected because the task titrates the go-stop offset on the fly in order to achieve an ~50% failure rate. Participants responded rapidly across all groups (C = 0.63s ± 0.22, R = 0.70s ± 0.23, P = 0.66s ± 0.20; $p > 0.5$). Similarly, the time taken to cancel a routine response (SSRT) did not differ significantly across groups (C = 0.27s ± 0.10, R = 0.23s ± 0.12, P = 0.22 ± 0.26; $p > 0.4$). Overall, the behavioural measures did not highlight any significant differences in performance between the three groups.

Table S1. Behavioural results, reporting mean values ± standard deviation. Time measurements are in seconds. df = Degrees of freedom; RT = response time; SSRT = Stop-Signal Reaction Time.

	Patients	Relatives	Controls	Statistics	df	p
Responses	174.6 ± 7.19	179 ± 9.68	174.3 ± 5.96	F = 2.14	2	0.1274
Mean RT (s)	0.66 ± 0.20	0.70 ± 0.23	0.63 ± 0.22	F = 0.53	2	0.5926
Mean go-stop offset (s)	0.48± 0.37	0.55 ± 0.33	0.43 ± 0.28	F = 0.62	2	0.5424
SSRT (s)	0.22 ± 0.26	0.23 ± 0.12	0.27 ± 0.10	F = 0.50	2	0.6091

Figure S1. Between group differences in brain activation focusing on peak co-ordinates of the F test of group main effect. Brain activation (y-axis) in the Lateral Occipital Complex (LOC), for each group (x-axis). ROI was generated by fitting 5mm radius spheres at peak F test coordinates in each hemisphere. Post hoc tests (permutation tests, 10,000 iterations) showed: activation in left and right LOC was higher in patients than controls (both $p<0.0001$); and was higher in patients than relatives ($p=0.0008$, $p=0.004$); whereas activation did not differ between relatives and controls (both $p>0.10$).

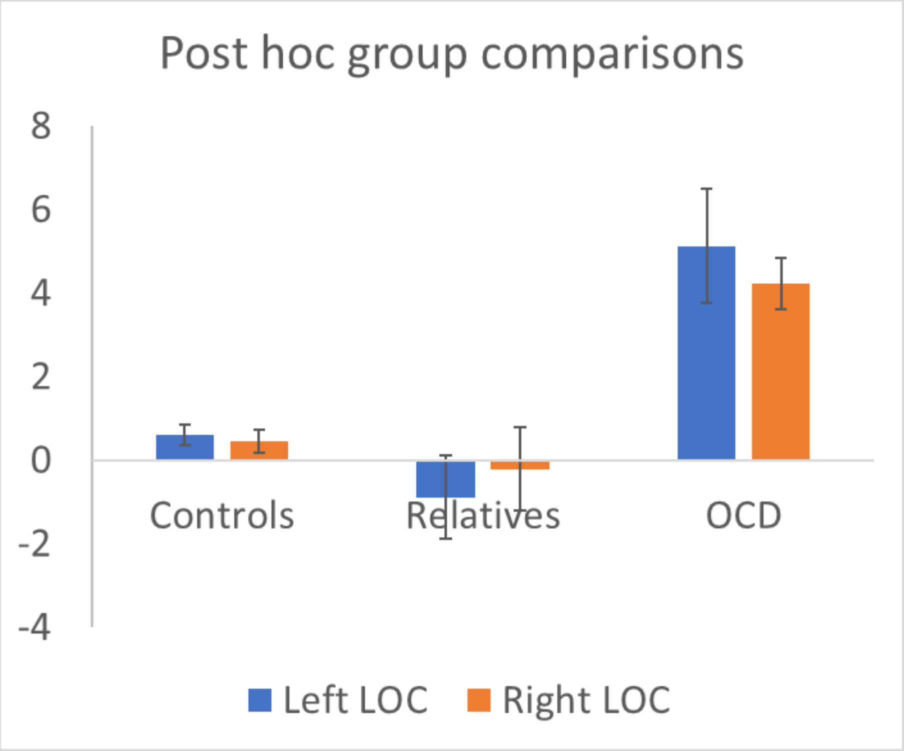

Table S2 – Post hoc analysis using ROIs from de Wit et al., AJP, 2012. At the request of a reviewer we undertook post hoc analysis using ROIs defined from this previous paper. We conducted MarsBaR ROI analyses with 10mm radius spherical ROIs as defined in the de Wit article. We observed significant activity throughout much of that ROI set during stop trials. There were no differences for successful-unsuccessful stop trials in any ROIs. There also were no cross group differences for stop trials in general, or for the group * stop/fail interaction. We were not able to replicate the De Wit results. We did not detect any significant group differences in these ROIs.

**Activity during stop trials collapsed across groups**				
	ROI	con	t	p(one tailed)
	ACC_sphere_10-0_20_34	3.89	2.18	0.015246
	L_IFG_sphere_10--33_23_-8	2.87	2.14	0.016778
	L_IPC_sphere_10--51_-55_43	9.13	5.25	0
	L_PSMA_sphere_10--15_14_67	1.83	1.39	0.083671
	R_IFG_sphere_10-33_23_-11	2.89	2.12	0.01755
	R_IPC_sphere_10-42_-55_43	7.22	3.72	0.000124
	R_PSMA_sphere_10-9_17_67	3.27	2.14	0.016732
	SN_sphere_10-3_-15_-2	-5.74	-3.25	0.999346
**Stop - fail collapsed across groups**				
	ROI	con	t	p(one tailed)
	ACC_sphere_10-0_20_34	0.49	0.57	0.284737
	L_IFG_sphere_10--33_23_-8	-0.53	-0.81	0.791102
	L_IPC_sphere_10--51_-55_43	-0.6	-0.71	0.761939
	L_PSMA_sphere_10--15_14_67	-0.63	-0.99	0.839582
	R_IFG_sphere_10-33_23_-11	-0.8	-1.22	0.888168
	R_IPC_sphere_10-42_-55_43	-0.95	-1.01	0.843543
	R_PSMA_sphere_10-9_17_67	0.45	0.61	0.272717
	SN_sphere_10-3_-15_-2	-1.38	-1.62	0.946879
**Control - other groups for all stop signal conditions**				
	ROI	con	t	p(one tailed)
	ACC_sphere_10-0_20_34	2.65	1.03	0.151145
	L_IFG_sphere_10--33_23_-8	1.82	0.94	0.173644
	L_IPC_sphere_10--51_-55_43	2.21	0.88	0.188757
	L_PSMA_sphere_10--15_14_67	-0.23	-0.12	0.548414
	R_IFG_sphere_10-33_23_-11	2.35	1.2	0.115111
	R_IPC_sphere_10-42_-55_43	1.43	0.51	0.304447
	R_PSMA_sphere_10-9_17_67	-0.53	-0.24	0.594954
	SN_sphere_10-3_-15_-2	1.58	0.62	0.266734
**Group * stop/fail interaction**				
	ROI	con	F	p(two tailed)
	ACC_sphere_10-0_20_34	0.59	0.14	0.873253
	L_IFG_sphere_10--33_23_-8	0.11	0.05	0.955509
	L_IPC_sphere_10--51_-55_43	1.12	0.27	0.76299
	L_PSMA_sphere_10--15_14_67	0.4	0.17	0.847088
	R_IFG_sphere_10-33_23_-11	3.92	1.55	0.215156
	R_IPC_sphere_10-42_-55_43	6.43	1.26	0.286989
	R_PSMA_sphere_10-9_17_67	0.91	0.28	0.753127
	SN_sphere_10-3_-15_-2	3.14	0.74	0.478323
